# Patient-centered nutrition education improved the eating behavior of persons with uncontrolled type 2 diabetes mellitus in North Ethiopia: a quasi-experimental study

**DOI:** 10.3389/fnut.2024.1352963

**Published:** 2024-04-10

**Authors:** Hagos Amare Gebreyesus, Girmatsion Fisseha Abreha, Sintayehu Degu Beshirie, Merhawit Atsbha Abera, Abraha Hailu Weldegerima, Afework Mulugeta Bezabih, Tefera Belachew Lemma, Tsinuel Girma Nigatu

**Affiliations:** ^1^Department of Nutrition and Dietetics, Jimma University, Jimma, Ethiopia; ^2^College of Health Sciences, Mekelle University, Mekelle, Ethiopia; ^3^Department of Pediatrics and Child Health, Jimma University, Jimma, Ethiopia

**Keywords:** patient-centered, nutritional education, eating behavior, uncontrolled type 2 diabetes mellitus, Ethiopia

## Abstract

**Background:**

Improving the clinical outcome of people with type 2 diabetes mellitus by modifying their eating behavior through nutrition education is an important element of diabetes self-management. Significant data from the literature supports this idea, however in the Ethiopian setting, there is a practice gap. Therefore, the purpose of this study was to assess how patient-centered nutrition education affected the eating behavior and clinical outcomes of people with uncontrolled type 2 diabetes mellitus.

**Method:**

In this quasi-experimental trial, 178 people with uncontrolled type 2 diabetes were purposely assigned to the intervention (*n* = 89) or control (*n* = 89) arm. The intervention arm was given patient-centered nutrition education, whereas the control arm received the routine care. Eating behavior and clinical outcome indicators such as HbAc, lipid profile, anthropometric indices, and blood pressure were assessed in both groups at the start and completion of the intervention. All scale variables were tested for normality and log transformed when appropriate. The baseline characteristics of the intervention and control groups were compared using the *t*-test for continuous variables and the chi-square test for categorical variables. The effect of nutrition education was determined using a difference in differences (DID) approach. *P* < 0.05 was established as the criterion of significance.

**Result:**

Food selection (DID = 15.84, *P* < 0.001), meal planning (DID = 31.11, *P* < 0.001), and calorie needs (DID = 37.65, *P* < 0.001) scores were statistically higher in the nutrition education arm. Furthermore, their overall eating behavior score (DID = 27.06, *P* < 0.001) was statistically greater than the controls. In terms of clinical outcomes, the overall picture reveals that the intervention did not outperform over the routine care. However, in comparison to the controls, the intervention arm showed clinically significant improvement in HbA1c (DID = −0.258, *P* = 0.485).

**Conclusion:**

Patient-centered nutrition education has resulted in positive adjustments in the eating behavior of people with uncontrolled type 2 diabetes mellitus. Furthermore, it has shown a great potential for improving their glycemic control.

## Background

Nearly entire diabetes care is accomplished by patients outside of a healthcare setting (1). Therefore, patient empowerment should be the main emphasis of initiatives meant to enhance diabetes care (2). The most important, yet complicated and difficult, of them is promoting and supporting adherence to a healthy diet (3). People with type 2 diabetes mellitus (T2DM) have a poor understanding of the role of nutrition in diabetes management (4). Since knowledge acquisition alone cannot result in behavioral change, even those who comprehend it have difficulty adapting their eating behavior to the new recommendation (5). As a result, unlike improvements in other facets of self-care, the involvement of people with T2DM in healthy eating is rather poor. Such a lack of awareness, and the resulting unhealthy eating behavior, leads to poor clinical prognosis and serious health implications (3).

Patient-centered nutrition education is a viable strategy to bridging the gap between nutrition information acquisition and eating behavior adjustment (6, 7). The teaching is adapted to the receivers’ needs, values, and preferences (8), as well as their food literacy and numeracy (9). As a result, it promotes knowledge, perception, and behavior of healthy eating (10, 11). Indeed, data revealed that patient-centered education enhanced patients’ satisfaction with their nutrition care (12) and helped them implement changes in eating behavior (13). However, implementation is difficult, and there is a considerable knowledge-to-practice gap (14).

Diabetes nutrition education services in general, and patient-centered approaches in particular, are scarce in Ethiopia (15–17). According to the American Diabetes Association, diabetic individuals should get healthy dietary pattern based nutritional counseling at diagnosis and as needed throughout their lives (18). However, actual information from most diabetes clinics shows that consultation periods are relatively short, leaving little or no opportunity for patient education. Furthermore, contrary to the ADA’s guideline (18), most facilities do not include a dietician/nutritionist as a member of the diabetes care team, and the service is frequently provided by physicians or nurses with insufficient nutrition expertise (19–25). This type of teaching may not provide the diabetic with the knowledge, skills, and motivation needed to achieve optimal healthy eating behavior (24, 25). As a result, the goal of this study was to assess how patient-centered nutrition education guided by the revised Pender’s Health Promotion Model (HPM) affected eating behavior and clinical outcomes in people with uncontrolled T2DM (HbA1c ≥ 7%).

## Materials and methods

**Study setting and participants:** The study was conducted in two General Hospitals namely Mekelle general Hospital (intervention area) and Adigrat General Hospital (control area) in Tigray region, North Ethiopia. Study participants were persons with uncontrolled T2DM who had follow up at each hospital.

**Study design:** A quasi-experimental design with a non-equivalent control group and a pretest-posttest format was used to assess the effect of patient-centered nutrition education on eating behavior and clinical outcomes of people with uncontrolled T2DM.

### Eligibility criteria

*Inclusion criteria:* We enrolled people with uncontrolled T2DM (HbA1c ≥ 7%), who were at least 18 years old, lived in the study area, could read Tigrigna (the local language), or who had a literate family member in the household, and owned a telephone (mobile or landline in the household).

*Exclusion criteria:* We excluded individuals with uncontrolled T2DM who had documented cognitive impairment, pregnant or nursing women, those who did not reside in the study area during the study period, and those who had changed medications in the most recent follow-up.

**Sample size:** The sample size was determined by placing the margin of error at 0.05, the power at 95%, and the intervention to control ratio at 1:1. The goal of the intervention was to effect a net reduction in HbA1c of 0.25 from the baseline (26). Assuming 15% attrition, there were 89 participants in each arm of the trial. The following formula was used to calculate the sample size:


$$\text{n = }\frac{\text{2}\left({\text{S}}^{\text{2}}\right){\left({\text{z}}_{\text{α/2}}{\text{ + z}}_{\text{β}}\right)}^{\text{2}}}{{\left(\text{Δμ}\right)}^{\text{2}}}$$

Where n is the sample size in each group; Z_α/2_, margin of error; Z_β_, the power; Δμ, the mean difference between the intervention and comparison groups, and S, the standard deviation from previous study.

**Sampling technique:** The two hospitals were randomly assigned to one of two groups: control or intervention. Following that, the experiment was available to anyone with uncontrolled T2DM who had been identified from the intervention hospital in a previous study and met the inclusion criteria. In the previous study, 324 patients from both institutions were classified as having uncontrolled T2DM. 250 of them volunteered to take part in the current study. Based on the inclusion criteria, obtaining a matching participant from the control group, and the calculated sample size, 89 persons were chosen from the intervention hospital to participate in the trial. An equal number of participants were picked from the control area to meet the 1:1 ratio, bringing the total sample size to 178.

**Outcomes:** Changes in eating behavior and HbA1c were the primary outcomes of the study. Changes in serum lipid profile, anthropometric indices, blood pressure and atherogenic index of the plasma were secondary outcomes.

**Hypothesis:** We hypothesized that a patient-centered NE intervention tailored to the participants’ needs, values, and preferences would result in significant improvements in the outcomes under investigation at 3 months.

**Intervention:** The intervention group received two patient-centered nutrition education sessions over the course of 3 months, separated by 1-month intervals. The education was delivered in a mixed approach. The initial education session lasted 30 min and was tailored to each participant. This was offered at the time of enrollment of the participants into the intervention. The content of the education was basic information about diabetes, its symptoms and classification; complications associated with T2DM and an introduction to diabetes self- management (DSM) modalities. In addition individual nutrition related concerns were discussed. It was given by the principal investigator, who is a PhD student in Human Nutrition, together with their physician, who was an internist. A month after the first session, a second group-based nutrition education session was offered by the principal investigator. This session took 120 min with a 10-min health break in the middle. Families and caregivers of participants’ were encouraged to attend, but only few have done so. Discussions regarding the contents of healthy diet (Choosing whole foods over highly processed ones, emphasizing use of non-starchy vegetables, fruits, minimizing refined grains, and avoiding added sugars, sugar-sweetened beverages, and trans fats); components of healthy eating (appropriate food selection, meal planning and identifying calorie needs); the objectives of healthy eating behavior (maintaining a healthy weight, acquiring nutritional requirements, achieving glycemic targets and other metabolic goals, and avoiding or reducing the progression of complications); and the methods for implementing healthy eating behavior were all included in the education. Planning meals and controlling portions were taught using the plate approach. A brochure with a brief summary of the second educational session was also distributed as a take-home guide for healthy eating.

With the contents specifically targeted to the participants’ eating behavior determinants discovered in a prior qualitative study, notably dietary knowledge, cost and availability of health foods, and social support, the patient-centered nutrition education was informed by the behavior-specific constructs of Pender’s HPM. The importance of healthy diet for all family members, regardless of their diabetes status, and meal planning on a limited budget were emphasized. The content was presented through Power Point presentations, discussions, and demonstrations. To help the subjects visualize portion sizes, the session used household measurements and food photographs. Participants’ relevant positive deviant eating behaviors were also deliberated. The PI also got participant phone numbers to facilitate follow-up. Participants were phoned to remind them to attend the second nutrition education session and to encourage them to follow the healthy eating guide.

**Compliance:** Monitoring participant attendance at training sessions and keeping track of who received the two-page pamphlet summarizing the training were two ways to gauge compliance with the intervention. Additionally, repeated phone calls were made to the participants to motivate them to follow the instructions for eating healthy.

### Data collection instruments and measures

Measurements were taken at the start and end of the trial. The baseline data were gathered prior to the intervention. Endline data were gathered right away following the closure of the intervention. The information collected, the tools utilized and the measurement techniques applied in both surveys were the same. Only at the baseline were demographic, socioeconomic, comorbidity status, and other pertinent clinical histories gathered.

A structured questionnaire created by the study team was used to gather demographic and clinical data (Supplementary Tables 1, 2). A Demographic Health Survey (DHS) instrument was used to gather household socioeconomic data (27) (Supplementary Table 3). The WHO Stepwise Approach (STEPS) instrument (28) and the Global Physical Activity Questionnaire (GPAQ) (29) provided the framework for the collection of anthropometric and physical activity data, respectively. The Charlson’s Comorbidity Index chart (30) was used to retrieve comorbidity information from their medical record. The Pentra C 400 clinical chemistry analyzer was used to do biochemical analysis in accordance with standard operating procedures (SOPs). While Humameter A1c was utilized to measure the HbA1c level.

Data on eating behavior was collected using a tool developed by Bhutanese investigators and customized to the setting by the research team (31, 32) (Supplementary Table 4). The Bhutanese tool had 19 Likert-type items. Based on the rigorous psychometric evaluation, 14 of the 19 items were validated for assessing the eating behavior of the participants in our setting. Of these, five items were used to assess the food selection dimension of the eating behavior. Six items were utilized to assess the meal planning dimension and the remaining three items were for calorie needs recognition dimension. Likert-type items in each dimension had four response anchors: strongly disagree, disagree, agree and strongly agree. They were given equal weight and allotted equidistant points that range from 1 for strongly disagree to 4 for strongly agree.

Each eating behavior dimension’s Likert-type item scores were added up to create Likert-scale scores. For ease of comparison with studies that used different numbers of Likert-items or responses, the raw scores were converted to percentage of scale to maximum score (%SM) (33). The percent SM range was set to 0 to 100 because the lowest possible score for our Likert-type item was one. An SM cutoff score of 66.7% was used to define participant eating behavior in each dimension as healthy or unhealthy. Participants with % SM ≥ 66.7 were deemed to practice healthy eating, whereas those with % SM < 66.7 were assumed to practice unhealthy eating. Ranks obtained in the three dimensions were aggregated to assess overall eating behavior. Our earlier article (32) provides a more detailed discussion of the tools and measurement methods.

### Statistical analysis

Data were twice entered into Epidata 3.1 (Xunta de Galicia, Spain & PAHO, USA), and then exported to Stata version 14SE for cleaning and analyses. The latent variable that best accounts for the socioeconomic variation among the participants was found using Principal Component Analysis (PCA). The relative socioeconomic status of the subjects was then determined based on their scores in the discovered latent variable. Normality was checked for all scale variables and log transformed when relevant. For continuous variables, unless otherwise stated, data were reported as means +/- SD, and for categorical variables, absolute frequencies and percentages were used. To predict mortality risk, an age-adjusted Charlson’s Comorbidity Index (CCI) was generated.

Baseline characteristics between intervention and control groups were compared using the *t*-test for continuous and chi-square test for categorical variables. Difference in differences (DID) analysis was carried out to test superiority of the patient-centered nutrition education package over the routine care with regard to improving eating behavior and clinical outcomes. Baseline variables (HDL & HDL/TC) demonstrating significant difference between the intervention and control groups were excluded from the DID analysis. The level of significance for all tests was set at *P* < 0.05.

### Ethical approval

The project (IHRPGD/467/2018) was reviewed and approved by the Institutional Review Board (IRB) of the Institute of Health at Jimma University. Health authorities at the regional and facility levels were informed of the study’s purpose; as a result, written authorization to commence was obtained. Potential participants were given information about the study, including how the data collected would be used. They received a briefing on the process for gathering the data and assurances that there would be no costs or risks involved. Furthermore, the confidentiality of the data and the freedom to withdraw from the study at any time without penalty were assured. Inclusion in the intervention or comparison arm was limited to participants who gave a written informed consent. Participants in the control arm were also informed, for ethical reasons, of the significance of physical activity for glycemic control.

## Results

### Subjects and baseline characteristics

170 of the initial 178 participants had finished the research and were taken into account in the final analysis. Four intervention group participants dropped out for personal reasons; three skipped the group-based nutrition education session and the rest one participant failed to pick up the brochure for a healthy eating guide. Likewise, four members of the control group were unable to participate in the endline survey and were consequently omitted from the final analysis. For the intervention arm, the average length of time with T2DM was 6.98 (±5.52) years, whereas for the control arm, it was 6.72 (±5.28) years. Their mean ages were 55.64 (±10.59) years for the control arms and 54.36 (±9.31) years for the intervention. For the intervention, the proportion of males and females was 42.4% and 57.6%, but for the control arms, it was 47.1% and 52.9%, Table 1. The baseline characteristics of the intervention and control group did not significantly differ from one another, as shown in Table 2. Furthermore, none of the baseline variables assessed indicated that the eight dropouts were different from the rest of the group.

**TABLE 1:** Comparison on socio-demographic, economic and clinical characteristics of study participants.

		Study group				
Characteristic		Intervention	Control	Mean diff.	X^2^(df)	Significance (p.value)
Mean age		54.36(±9.31)*	55.64(±10.59)*	−1.271		0.41
Sex	Male	36	40			
	Female	49	45		0.381(1)	0.537
Marital status	Single	10	13			
	Married	48	47			
	Divorced	6	7		0.710(3)	0.871
	Widowed	21	18			
Educational status	Illiterate	28	36			
	Able to read and write	13	12			
	1° level	10	14		4.707(4)	0.319
	2° level	13	6			
	College and above	21	17			
Economic status	Poorer	13	18			
	Poor	14	17			
	Average	13	19		1.312(4)	0.859
	Wealthy	17	15			
	Wealthiest	15	16			
Antidiabetic drug	Metformin	15	18			
	Glibenclamide	12	5			
	Metformin and glibenclamide	38	50		6.791(3)	0.079
	Insulin	20	12			
Treatment adherence	High	74	74			
	Moderate	11	10		1.048(2)	0.592
	Low	0	1			
Physical activity status	≥600 MET-min/week	57	63			
	<600 MET-min/week	11	9		1.033(2)	0.597
	Sedentary	17	13			
Mean duration of DM (years)		6.98(±5.52)*	6.72(±5.28)*	0.257		0.756
Mean CCI		2.56(±1.22)*	2.44(±1.19)*	0.124		0.503

X^2^ – chi-square, df, degree of freedom; CCI, Charlson’s Comorbidity Index, *- mean.

**TABLE 2:** Comparison of baseline clinical, biochemical and anthropometric variables.

Variable	Comparison X ± SD	Intervention X ± SD	Mean difference	P.value
HbA1c (%)	9.324 (1.704)	9.206 (1.653)	-0.118	0.652
Total Cholesterol (mg/dl)	185.851 (45.79)	178.423 (42.86)	-7.427	0.275
LDL Cholesterol(mg/dl)	98.013 (31.65)	94.541 (29.27)	-3.472	0.515
HDL Cholesterol (mg/dl)	36.829 (11.12)	40.654 (10.96)	3.825	0.036
Triglyceride (mg/dl)	156.932 (89.10)	143,081 (82.21)	-13.851	0.277
TC to HDL ratio	5.349 (1.61)	4.581 (1.26)	-0.769	0.000
Atherogenic index	0.585 (0.304)	0.494 (0.310)	-0.091	0.053
Waist circumference (cm)	87.624 (12.31)	90.612 (11.65)	2.988	0.096
BMI (kg/m^2^)	22.843 (3.65)	22.829 (3.67)	-0.015	0.979
Waist to hip ratio	0.899 (0.11)	0.922 (0.08)	0.024	0.244
Waist to height ratio	0.533 (0.08)	0.553 (0.07)	0.020	0.063
Systolic blood pressure (mmHg)	128.75 (18.23)	127.14 (18.56)	-1.612	0.564
Diastolic blood pressure (mmHg)	81.19 (10.76)	80.28 (11.39)	-0.906	0.587

X ± SD-mean ± standard deviation.

Table 3 summarizes the changes in the eating behavior dimensions from pre-intervention to post-intervention and compares the differences between the two groups. For any of the variables at the baseline, no differences between the two groups were found to be statistically significant. However, when the intervention was given, the intervention group showed statistically significant improvements. While the scores of the control group showed a non-significant decline. In terms of the difference in differences (DID) analysis between the two arms, the study found that the intervention arm’s overall eating behavior changed by 27 percentage points when compared to the controls (DID = 0.27, *P* < 0.000). The overall eating behavior in the intervention arm improved by 21 percentage points, while it was declining by roughly 6 percentage points in the control arm. The mean changes between the two groups were also found to differ in each of the eating behavior dimensions, with statistically significant differences being noted.

**TABLE 3:** Comparison of mean values of eating behavior dimensions between the intervention and comparison group.

Characteristic	Unstandardized mean scores								Standardized mean scores (%SM)							
	Comparison			Intervention			DID	P.value	Comparison			Intervention			DID	P.value
	Baseline	Endline	Difference	Baseline	Endline	Difference			Baseline	Endline	Difference	Baseline	Endline	Difference		
Food selection	13.65	13.75	0.1	13.60	16.08	2.48	2.38	0.00	57.65	58.35	0.7	57.33	73.88	16.55	15.84	0.00
Meal planning	11.12	9.39	−1.73	11.36	15.24	3.88	5.6	0.00	28.43	18.82	−9.61	29.80	51.31	21.51	31.11	0.00
Calorie recognition	5.27	4.22	−1.05	5.04	7.38	2.34	3.39	0.00	25.23	13.59	−11.6	22.61	48.63	26.02	37.65	0.00
Overall eating	30.04	27.36	−2.68	30	38.69	8.69	11.36	0.00	38.18	31.82	−6.36	38.10	58.80	20.7	27.06	0.00

Unstandardized mean scores denote the raw mean scores for each eating behavior dimension and the overall eating behavior; standardized mean scores denote that the means are transformed to a percentage of the scale to the maximum score (%SM).

Table 4 summarizes the changes in the clinical outcome indicator variables. Again, the two groups showed no statistically significant differences with regard to most of their baseline variables except for HDL and TC/HDL (data not shown). When compared to the control group, the mean HDL value in the intervention group was considerably higher (40.65 ± 10.96 vs. 36.83 ± 11.12, *P* = 0.036). In contrast, the mean TC/HDL in the intervention arm was considerably lower than in the control arm (4.58 ± 1.26 vs. 5.35 ± 1.61, *P* = 0.000). No clinical outcomes were significantly varied between the two arms as a result of the intervention. However, the intervention arm remarked within-group statistically significant reductions in HbA1c (delta *X* = −0.49, *p* < 0.001), as was seen for total cholesterol in both arms (C: −13.655, *P* = 0.004, I: −21.100, *P* < 0.001). In contrast, statistically noteworthy increments in LDL cholesterol (C; 21.51, *p* < 0.001, I; 13.33, *P* = 0.002) were evident among participants in either arm.

**TABLE 4:** Comparison of mean values of anthropometric, clinical, and biochemical variables between the intervention and comparison group.

	Comparison				Intervention					
Variable	Baseline	Endline	Diff	P.value	Baseline	Endline	Diff	P.value	DID	P.value
HbA1c	9.324	9. 087	-0.237	0.080	9.206	8.712	-0.494	0.000	-0.258	0.485
TC	185.851	172.195	-13.655	0.004	178.423	157.322	-21.100	0.000	-7.445	0.438
LDL-C	98.013	119.519	21.506	0.000	94.541	107.872	13.330	0.002	-8.176	0.279
Triglyceride	156.932	144.789	-12.142	0.105	143.081	128.286	-14.795	0.122	-2.653	0.883
AIP	0.585	0.495	-0.090	0.001	0.494	0.427	-0.067	0.072	0.023	0.733
WC	87.624	88.459	0.835	0.389	90.612	90.753	0.141	0.824	-0.694	0.784
BMI	22.843	22.629	-0.214	0.268	22.829	22.653	-0.176	0.439	0.038	0.960
WHtR	0.533	0.540	0.007	0.280	0.553	0.556	0.003	0.549	-0.004	0.799
Systolic BP	128.75	122.47	-6.282	0.003	127.14	127.07	-0.071	0.971	6.212	0.117
Diastolic BP	81.19	79.06	-2.129	0.141	80.28	80.35	0.071	0.954	2.200	0.351

Unstandardized mean scores denote the raw mean scores for each anthropometric, clinical and biochemical variables; standardized mean scores denote that the means are transformed to a percentage of the scale to the maximum score (%SM).

## Discussion

The study demonstrated that patient-centered nutrition education informed by Pender’s Health Promotion Model behavior-specific constructs promotes healthy eating behavior. However, there were no statistically supported changes in clinical outcomes, with the exception of within-group substantial reductions in HbA1c and total cholesterol.

Adoption and maintenance of healthy eating is a key strategy to improve glycemic control, minimize risk of complications and improve health of persons with diabetes (34). Importantly, people’s eating behaviors are influenced, among other things, by their perceptions and knowledge of a healthy diet (35). Therefore, in order to successfully promote and support healthy eating, nutrition education that communicates sufficient knowledge and encourages behavior modification is essential (36). Confirming this concept, participants in the intervention arm made significant improvements in their overall eating behavior compared to the control arm in the present study. Additionally, the intervention arm considerably outperformed the control arm across all dimensions of eating behavior. Similar to this study, a number of randomized controlled trials and systematic reviews have also noted the link between nutrition education and better eating behavior (37–40).

The principal favorable outcome anticipated as a result of improved eating behavior is better glycemic control (41, 42). This conclusion was supported by the UK prospective diabetes research (43), which found that after a 3-month dietary intervention, newly enrolled diabetic patients’ HbA1c levels significantly reduced (from 9.1 to 7.2%) (43). Additionally, a number of cohort and randomized control trials have shown how nutrition education can help people with glycemic control (37, 44–47). However, despite being remarkable, the reduction in HbA1c as a result of implementing patient-centered nutrition education in the current trial did not reach statistical significance. Other similar studies (8, 48) also reported encouraging improvements in HbA1c levels that did not achieve statistical significance.

Although the difference in HbA1c levels between the two arms was statistically small, the finding was functionally important. According to the literature, a 0.3 to 0.5 drop in HbA1c value is considered clinically significant (49, 50). In this study, the 3-month nutrition education lowered the mean HbA1c within the intervention arm by roughly 0.5%, which was well-matched to the literature report. As a result, despite its statistical insignificance, the intervention could be clinically relevant in lowering the risk of diabetes-related complications. According to the UK Prospective Diabetes Study (UKPDS), a 1% decrease in HbA1c reduces microvascular complications by 37% and diabetes-related mortality by 21% (51). If risk reduction is proportional to HbA1c reduction, then the current study would reduce the risk of microvascular complications by roughly 19% and fatalities by 11%.

In addition to a higher HbA1c, T2DM is linked to significant alterations in plasma lipid and its components. In persons with uncontrolled T2DM, increases in LDL, TC, TG, and a decrease in HDL are frequent conditions that lead to Coronary Heart Disease (CHD) (52). Besides, increasing blood pressure and unfavorably changing body composition indices are extremely evident in further escalating the CHD risk (53). Unlike pharmacologic treatment, dietary intervention is capable of avoiding all of these CHD risk factors and is regarded as a cornerstone of diabetes control (54). Various studies supporting this concept found that dietary education effectively improved serum lipids, systolic and diastolic blood pressure, BMI, and waist circumference (47, 52, 55, 56). In the current study, however, providing nutrition education for 3 months had no effect on any of the previously listed clinical variables. Consistent with this conclusion, multiple randomized control trials also failed to establish the effectiveness of nutrition education in changing lipid profile, blood pressure, and anthropometric indices (48, 57, 58). Though further research is needed to determine the exact reasons, differences in educational content, delivery method, length, and frequency of intervention may explain this disparity.

Favorable reductions in HbA1c are likely to succeed tangible changes in the participants’ eating behavior. Though a series of assessments were not done to determine the exact time required to adopt a healthy eating behavior, it is implied that a significant amount of time will elapse before the desired behavior alteration is achieved. As a result, the remaining time would be insufficient for biochemical changes to occur, limiting the achievement of significant reductions in HbA1c within the allocated time frame. This is demonstrated by a clinically significant reduction in HbA1c in the intervention arm, albeit not substantially superior to the control. This explanation also applies to the other clinical outcomes studied, as their improvement is thought to be dependent on suitable reductions in HbA1c. Further increase in LDL cholesterol during the course of the trial in both groups supports our view, as it demonstrates the inadequacy of the achieved glycemic reduction in triggering hormonal changes that reverse the lipogenesis process occurring in the liver. However, the reduction in atherogenic index of plasma (AIP) in both arms is noteworthy, which could be attributed to regulated fat consumption and/or adequate physical exercise.

### Strengths and limitations

The nutrition education package in this study is specifically designed to meet the needs and preferences of the participants, which is its main strength. As a limitation, the participants’ self-reported data were used to assess the eating behavior, and objective biomarkers of food consumption were not used to confirm it. Thus, the chance of social desirability bias is not completely ignored, so care must be taken while interpreting it. However, as the intervention package is tailored to the participants’ situation, the observed behavior changes are more likely to occur. In contrast, the short intervention period appears to unacceptably shorten the time between behavior alteration and subsequent biochemical changes. Thus, despite the fact that it appears likely that the intervention package will enhance the participants’ glycemic control, this conclusion is precluded by the issue of time.

## Conclusion

Patients with uncontrolled T2DM who received the patient-centered nutrition education intervention in this trial reported significant improvements in both specific eating behavior dimensions and overall eating behavior. In addition, the provided intervention showed a promising potential to lower the individuals’ HbA1c levels. Throughout the course of the trial, some of the secondary outcomes exhibited further derangement. This is likely explained by the fact that the intervention period ended before the beneficial metabolic effects of eating behavior change were ensued. Therefore, the time for measuring such clinical outcome indicators in future trials needs to be carefully considered. A patient center nutrition education initiative at the hospital level, with sufficient follow-up and training for health professionals to enhance their knowledge and skills, is advised to improve the eating behavior of persons with T2DM.

## Data availability statement

The raw data supporting the conclusions of this article will be made available by the authors, without undue reservation.

## Ethics statement

The studies involving humans were approved by the Institutional Review Board of Jimma University, Jimma, Ethiopia. The studies were conducted in accordance with the local legislation and institutional requirements. The participants provided their written informed consent to participate in this study.

## Author contributions

HG: Conceptualization, Data curation, Formal analysis, Funding acquisition, Investigation, Methodology, Project administration, Resources, Software, Supervision, Validation, Visualization, Writing – original draft, Writing – review and editing. GA: Funding acquisition, Methodology, Project administration, Resources, Supervision, Validation, Visualization, Writing – review and editing. AW: Methodology, Supervision, Validation, Visualization, Writing – review and editing. SB: Data curation, Investigation, Methodology, Supervision, Writing – review and editing. MA: Methodology, Supervision, Validation, Visualization, Writing – review and editing. AB: Methodology, Supervision, Validation, Visualization, Writing – review and editing. TL: Methodology, Supervision, Validation, Visualization, Writing – review and editing. TN: Conceptualization, Methodology, Supervision, Validation, Visualization, Writing – review and editing.
