# Supplementary material for: Patient-centered nutrition education improved the eating behavior of persons with uncontrolled type 2 diabetes mellitus in North Ethiopia: a quasi-experimental study
Source: Front Nutr. 2024 Apr 10;11:1352963. doi: 10.3389/fnut.2024.1352963 (PMC11040084; doi:10.3389/fnut.2024.1352963)
Supplement: Supplementary file 1 [file Table_1.docx]

1. **Demographic background of participants**

| **No** | **Question** | **Response** | | **Go to/Skip** |
| --- | --- | --- | --- | --- |
| 101 | Age |  | _____ |  |
| 102 | Sex | Male  Female |  |  |
| 103 | Nationality |  | ________ |  |
| 104 | Ethnicity | Tigrian  Amhara  Oromo  Afar  Other (specify) |  |  |
| 105 | Religion | Orthodox  Muslim  Catholic  Protestant  Others (specify) |  |  |
| 106 | Marital status | Single  Married  Widowed  Divorced |  |  |
| 107 | Educational status | Illiterate  Able to read and write  Primary school  Secondary school  College graduate or above |  |  |
