# Supplementary material for: Patient-centered nutrition education improved the eating behavior of persons with uncontrolled type 2 diabetes mellitus in North Ethiopia: a quasi-experimental study
Source: Front Nutr. 2024 Apr 10;11:1352963. doi: 10.3389/fnut.2024.1352963 (PMC11040084; doi:10.3389/fnut.2024.1352963)
Supplement: Supplementary file 2 [file Table_2.docx]

**2. Diabetic follow up, nutrition information and anthropometric and biochemical data of adults with type 2 diabetes mellitus**

| 201 | How long has it been since you diagnosed with T2DM? | ------- years |  |  |
| --- | --- | --- | --- | --- |
| 202 | How long has it been since you begun DM follow-up? | --------years |  |  |
| 203 | How many times do you visit the diabetic follow up clinic in the last six months? | -----------/year |  |  |
| 204 | How do you control your blood glucose? | By diet  By exercise  By medication  By diet and exercise  By diet and medication  By exercise and medication  By diet, exercise & medication |  |  |
| 205 | Type of medication for diabetes | Metformin  Glibenclamide  Metformin & Glibenclamide  Insulin  Insulin + Metformin | **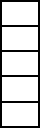** |  |
| 206 | Treatment adherence | Poor  Moderate  High | **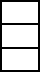** |  |
| 207 | Do you have other co morbidities or complications | Yes  No |  | If No skip 208 |
| 208 | What type? specify | Hypertension  Retinopathy  Neuropathy  Nephropathy  Cardiovascular diseases  Others (specify) |  |  |
| 209 | Did you get any nutritional education or advice regarding dietary control of diabetes so far? | Yes  No |  | If the response is No, skip Q210-211 |
| 210 | Who gave it to you and where?  (More than one response is allowed) | Doctor  Nurse  Nutritionist  Other team of health professionals as a community service |  |  |
| 211 | How was it given? More than one response is possible | Individually  In Group  Orally  In written |  |  |

**212. Anthropometric and biochemical data of persons with type 2 diabetes**

| Measurement | Value | Date | Measurement | Value 1 | Value 2 | Average | Date |
| --- | --- | --- | --- | --- | --- | --- | --- |
| HbA1c |  |  | Waist circumference (cm) |  |  |  |  |
| Total cholesterol |  |  | Height(m) |  |  |  |  |
| Triglyceride |  |  | Weight(kg) |  |  |  |  |
| LDL |  |  |  |  |  |  |  |
| HDL |  |  |  |  |  |  |  |

BMI……………… Waist-to-hip ratio…………

Blood pressure in __________ mmHg
