# Supplementary material for: Patient-centered nutrition education improved the eating behavior of persons with uncontrolled type 2 diabetes mellitus in North Ethiopia: a quasi-experimental study
Source: Front Nutr. 2024 Apr 10;11:1352963. doi: 10.3389/fnut.2024.1352963 (PMC11040084; doi:10.3389/fnut.2024.1352963)
Supplement: Supplementary file 3 [file Table_3.docx]

**3. Characteristics of house and household assets**

| **S.no** | **House characteristic or asset** | **Response options** | | | | **Go to/skip** | |
| --- | --- | --- | --- | --- | --- | --- | --- |
| 301 | Do you own your house or you are renting for? | 1= Own  2= Renting | | | |  | |
| 302 | What is the main material of your house’s roof? | 1 = thatch/grass or leaves  2 = iron sheets  3 = other…specify | | |  | | |
| 303 | What is the main material of your house’s walls? | 1 = no walls  2 = natural materials cane, wood, mud  3 = planks  4 = stone with mud  5 = stone with cement/bricks  6 = other | | |  | | |
| 304 | What is your house’s main floor material? | 1 = natural floor (earth/sand/dung)  2 = rudimentary floor (wood/palm/bamboo)  3 = finished floor (polished wood, vinyl, tiles, cement, carpet)  4 = other | | |  | | |
| 305 | What kind of toilet facilities does your household have? | 1= no facility/bush/field  2= pit toilet/latrine  3= Ventilated improved pit latrine  4= flush toilet  5=other | | |  | | |
| 306 | What is the main source of drinking water for members of your household? | 1=piped water into dwelling  2=piped water into compound  3=public tap  4=borehole in compound  5=dug well protected  6= dug well unprotected  7=water from protected spring  8= water from unprotected spring  9=tanker truck  10=surface water (river/dam/lake/pond/stream/ canal irrigation channel)  11=bottled water  Other (specify) ______ | | |  | | |
| 307 | What is the main source of water used by your household for other purposes such as cooking and hand washing? | 1=piped water into dwelling  2=piped water into compound  3=public tap  4=borehole in compound  5=dug well protected  6= dug well unprotected  7=water from protected spring  8= water from unprotected spring  9=tanker truck  10=surface water (river/dam/lake/pond/stream/ canal irrigation channel)  11=bottled water  Other (Specify) | | |  | | |
| 308 | Do you do anything to the water to make it safer to drink? | 1 = Yes  2 = No  3 = Don’t know | | |  | | |
| 309 | If yes to Q no 8, what is the main thing you do? | 1 = Let it stand and settle  2 = Strain through a cloth  3 = Use water filter (ceramic/sand/composite/etc.)  4 = Boil  5 = Solar disinfection  6 = Add bleach/chlorine  7 = Other  8 = Don’t know | | |  | | |
| 310 | What type of fuel does your household mainly use for cooking? | 1=electricity  2=LPG  3= Natural gas  4= Biogas  5=kerosene  6=charcoal  7=firewood/straw  8=dung  9=other  10= No food is cooked in the Household | | |  | | |
| 311 | Is the cooking usually done in the house, in a separate building, or outdoors? | 1= In the house  2= In a separate building  3= Outdoors  4= Other (specify) | | |  | | |
| 312 | Do you have a separate room which is used as a kitchen? | 1= Yes  2= No | | | If the response is Yes, go to Q 213 | | |
| 313 | Is the house connected to electricity? | 1= Yes  2= No | | |  | | |
| 314 | How many rooms in this household are used for sleeping? | _______ rooms | | |  | | |
| 315 | Does your household have the following home appliances? |  | Item | Yes | | | No |
|  |  | 15. 1 | A mobile phone |  | | |  |
|  |  | 15. 2 | A bajaj |  | | |  |
|  |  | 15. 3 | A refrigerator |  | | |  |
|  |  | 15. 4 | A non-mobile telephone |  | | |  |
|  |  | 15.5 | A television |  | | |  |
|  |  | 15.6 | A computer |  | | |  |
|  |  | 15.7 | A table |  | | |  |
|  |  | 15.8 | A chair |  | | |  |
|  |  | 15.9 | A bed with cotton/sponge/spring mattress |  | | |  |
|  |  | 15.10 | An electric pan |  | | |  |
|  |  | 16.11 | A kerosene lamp/pressure lamp |  | | |  |
| 316 | Does this household own any livestock, herds, other farm animals, or poultry? |  | 1= Yes  2= No |  | | | If the response is No, go to Q no.216 |
| 317 | How many of the following animals do this household own? |  | Type of livestock | 0 = If not available | | | Put exact number if available |
|  |  |  | Milk cows, oxen or bulls? |  | | |  |
|  |  |  | Other cattle? |  | | |  |
|  |  |  | Horses, donkeys, or mules? |  | | |  |
|  |  |  | Camels |  | | |  |
|  |  |  | Goats? |  | | |  |
|  |  |  | Sheep? |  | | |  |
|  |  |  | Chickens or other poultry? |  | | |  |
|  |  |  | Beehives? |  | | |  |
| 318 | Does any member of this household own any agricultural land? | 1= Yes  2= No | | If the response is no, go to Q 220 | | | |
| 319 | If yes for Q 218, how many hectares of agricultural land do members of this household own? | _________ Hectars | |  | | | |
| 320 | Does any member of this household have a bank account? | 1= Yes  2= No | |  | | | |
