# Supplementary material for: Patient-centered nutrition education improved the eating behavior of persons with uncontrolled type 2 diabetes mellitus in North Ethiopia: a quasi-experimental study
Source: Front Nutr. 2024 Apr 10;11:1352963. doi: 10.3389/fnut.2024.1352963 (PMC11040084; doi:10.3389/fnut.2024.1352963)
Supplement: Supplementary file 4 [file Table_4.docx]

**4. Eating behavior questionnaire (food selection, meal planning and calorie needs recognition)**

| **Eating behavior dimensions and items** | | **Response** | | | |
| --- | --- | --- | --- | --- | --- |
|  |  | Strongly disagree | Disagree | Agree | Strongly agree |
| **Food selection dimension** | |  |  |  |  |
| 401 | You choose foods that contain low to medium glycemic index in your diet |  |  |  |  |
| 402 | You or the person who cooks for you rarely uses saturated fats for cooking |  |  |  |  |
| 403 | You eat fruits and vegetables every day |  |  |  |  |
| 404 | You avoid salty diet |  |  |  |  |
| 405 | You avoid or take alcohol in moderation |  |  |  |  |
|  | **Meal planning dimension** |  |  |  |  |
| 406 | You understand and able to arrange your right meal plan |  |  |  |  |
| 407 | You understand and able to use plate methods in arranging your meal plan within a day as follows:   - 1^1^/_2_ plate of vegetables - A plate of meat or meat substitutes - A plate of starch - 3 glasses of milk/fruit or 1 fruit - Oils or butter sparingly |  |  |  |  |
| 408 | You understand and able to use food exchange list in arranging your meal plan |  |  |  |  |
| 409 | You eat 3 meals and 3 snacks a day |  |  |  |  |
| 410 | You eat meal in the same time every day |  |  |  |  |
| 411 | You eat a variety of foods in every meal daily that include the following  -Fleshy foods (fish, chicken or meat)  - Cereals (Teff, wheat, barley)  - Low fat  - Vegetables (Spinach, Lettuce, Cabbage)  - Fruits (Orange, Mango, Papaya)  - Pulses (Beans, Peas, lentils) |  |  |  |  |
|  | **Calorie needs recognition dimension** |  |  |  |  |
| 412 | You know and maintain the calorie proportions you should take in each meal from:  - Carbohydrate  - Protein  -Fat |  |  |  |  |
| 413 | You weight and measure calorie of food in each meal using cups, grams or serving sizes |  |  |  |  |
| 414 | You consume same amount of food every day |  |  |  |  |
